# A New Ultrasound Biomicroscopic Sign seen after Deep Sclerectomy (Dolphin Head Sign)

**DOI:** 10.5005/jp-journals-10008-1202

**Published:** 2016-08-05

**Authors:** Ahmed M Abdelrahman, Hala M El Cheweikh, Dina MS El-Fayoumi, Riham SHM Allam

**Affiliations:** 1 Professor, Department of Ophthalmology, Kasr Al-Ainy School of Medicine, Cairo University, Giza, Egypt; 2 Professor, Department of Ophthalmology, Kasr Al-Ainy School of Medicine, Cairo University, Giza, Egypt; 3 Lecturer, Department of Ophthalmology, Kasr Al-Ainy School of Medicine, Cairo University, Giza, Egypt; 4 Lecturer, Department of Ophthalmology, Kasr Al-Ainy School of Medicine, Cairo University, Giza, Egypt

**Keywords:** Deep sclerectomy, Dolphin head sign, Glaucoma, Glaucoma surgery, Ultrasound biomicroscopy.

## Abstract

**Purpose:** To describe a new ultrasound biomicroscopic (UBM) sign seen in patients who underwent deep sclerectomy (DS) as a surgical procedure for the management of uncontrolled primary open-angle glaucoma (POAG). The presence of this sign in ultrasound biomicroscopy is suggested to be an indicator of successful surgery. We would like to name this sign as the “dolphin head sign.”

**Design:** Prospective interventional study.

**Materials and methods:** Twenty-eight eyes of 17 patients with POAG underwent DS with intraoperative mitomycin C (MMC) 0.3% applied for 2 minutes under the superficial scleral flap. Patients were followed up for a minimum of 6 months. Ultrasound biomicroscopy was done at the third postoperative month to evaluate the surgical area in both successful and failed cases.

**Results:** The study included 28 eyes of 17 patients. The mean age of the study group was 42.90 ± 14.37 years (20–64 years). The study included 10 females and 7 males. The mean preoperative intraocular pressure (IOP) was 24.57 ± 6.37 mm Hg (20-38 mm Hg). The mean best corrected visual acuity (BCVA) was 0.57 ± 0.3 (0.05–1.00). Complete success has been achieved in 21 eyes (75%) during the follow-up period, with a mean IOP of 12.00 ± 3.86 mm Hg (6–20 mm Hg). The dolphin head sign was demonstrated only in successful cases, whereas the unsuccessful cases failed to show the typical sign.

**Conclusion:** The presence of a “dolphin head” configuration in UBM images could be taken as an indicator of successful DS.

**How to cite this article:** Abdelrahman AM, El Cheweikh HM, El-Fayoumi DMS, Allam RSHM. A New Ultrasound Biomicroscopic Sign seen after Deep Sclerectomy (Dolphin Head Sign). J Curr Glaucoma Pract 2016;10(2):56-59.

## INTRODUCTION

In the 1990s, nonpenetrating glaucoma surgery (NPGS) like viscocanalostomy and deep sclerectomy (DS) were introduced as safer alternatives to trabeculectomy.^1,2^ The main difference between NPGS and trabeculec-tomy is the creation of a filtration membrane: The trabeculo-Descemet’s membrane (TDM) instead of a sclerostomy. Excision of the inner scleral flap during NPGS will create a subscleral lake (intrascleral bleb) where the aqueous collects. The postsurgical outflow pathways of aqueous remain unclear, but increased flow through the Schlemm’s canal, suprachoroidal space, and subconjunctival flow with “bleb” formation have all been postulated.^3-6^ Most reports agree that NPGS has a lower rate of complications than trabecu-lectomy.^7-9^ Deep sclerectomy has been shown to be of equal results to trabeculectomy in lowering the intraocular pressure (IOP).^10,11^ Similarly to trabeculectomy, intraoperative mitomycin C (MMC) application results in lower IOP.^12^

Ultrasound biomicroscopic (UBM) studies in human eyes that underwent DS have shown formation of a subscleral lake, an overlying bleb, and a supraciliary hypoechoic area.^13^

In our study, we noticed that the UBM appearance of the surgical area simulates the “dolphin head” in successful cases. Accordingly, we named the configuration of the surgical area as the “dolphin head sign.”

The aim of the present work is to describe this new sign seen with UBM during evaluation of eyes with DS and to correlate its presence with surgical success of the cases.

## MATERIALS AND METHODS

### Study Subjects

This is a prospective interventional study including 28 eyes of 17 patients having primary open-angle glaucoma (POAG) for which DS was done. The study subjects were recruited from Kasr Ainy subspecialty glaucoma clinic in the period starting from November 2013 to March 2014. The patients were followed up for a minimum period of 6 months. They were enrolled in this study after giving a written informed consent to participate and the Tenets of the Declaration of Helsinki were followed.

Deep sclerectomy was performed under the standard peribulbar anesthesia. Fornix-based conjunctival incision was done followed by a 4 × 3 mm rectangular superficial scleral flap using a crescent blade. Dissection was done until we got adequate exposure of the corneal periphery. Mitomycin C soaked sponges (0.3% for 2 minutes) were applied under the superficial scleral flap and the conjunctiva. Deep flap dissection was then carried out with deroofing of Schlemm’s canal. Percolation was then tested using sponges and the deep flap was excised. The superficial flap was then sutured using 10/0 nylon sutures one at each corner of the flap. The whole operations were performed by a single experienced surgeon (AA).

The patients were followed up for the IOP, best corrected visual acuity (BCVA) as well as full ophthalmo-logical assessment. Ultrasound biomicroscopy was done in the third postoperative month to evaluate the surgical area and compare its appearance to IOP control.

Ultrasound biomicroscopy was done by a single experienced investigator (HE) using a UBM 50 MHz, Ophthalmic Technologies Inc; Canada (OTI) ultrasonic scan images. With the patient in the supine position, and with the aid of an emersion scleral shell and an examination gel, the surgical area was scanned with the UBM probe. Radial and transverse sections of the sclerectomy area at 12 o’clock were explored using optimal dB gain that allowed best resolution and quality of images.

Statistical analysis was performed using Statistical Package for the Social Sciences (SPSS) for Windows (SPSS Inc, Chicago, Illinois, USA). The statistical data were expressed in terms of mean, range, and statistical deviation.

## RESULTS

Our study was performed on 28 eyes of 17 patients who underwent DS with MMC between November 2013 and March 2014 at the Department of Ophthalmology, Kasr Al-Ainy School of Medicine, Giza, Egypt.

**Figs 1A to C:**
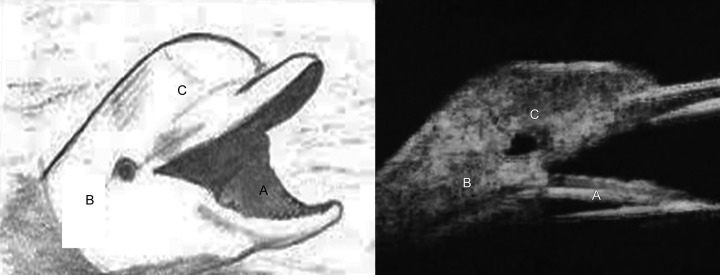
The head of the dolphin (left) compared to the ultrasound biomicroscopic appearance in deep sclerectomy (right): (A) Mouth of the dolphin compared to anterior chamber angle in UBM; (B) eye of the dolphin compared to intrascleral lake; and (C) head of the dolphin compared to filtering bleb

**Table 1:** **Table 1:** Preoperative and demographic data of successful cases

		*Range*		*Mean±SD*	
Age		20-64 years		42.90 ± 14.37	
Gender		7 F:5 M		58.3%:41.6%	
Preoperative medications		1-4		2.9 ± 0.2	
Preoperative IOP		20-38 mm Hg		24.57 ± 6.37 mm Hg	
BCVA		0.05-1.00		0.57 ± 0.3	
OD/OS		11 OD:10 OS		52.4%:47.6%	

All patients underwent DS with the adjuvant use of MMC (0.3% applied for 2 minutes), and the patients were followed up for a period of 6 months during which a UBM was performed in the third postoperative month to assess the surgical area.

During the study period, 21 eyes (75%) achieved IOP control without the need for adjuvant therapy (complete success; IOP < 21 mm Hg without medications or interventions) with a mean IOP value of 12.00 ± 3.86 mm Hg (6–20 mm Hg) (Table 1).

Seven eyes (25%) of five patients did not show criteria of surgical success with persistently high IOP above 22 mm Hg (mean IOP value of 24.8 ± 0.73 mm Hg) that required control using two topical antiglaucoma medication in four eyes and subscleral trabeculectomy in one eye.

**Fig. 2:**
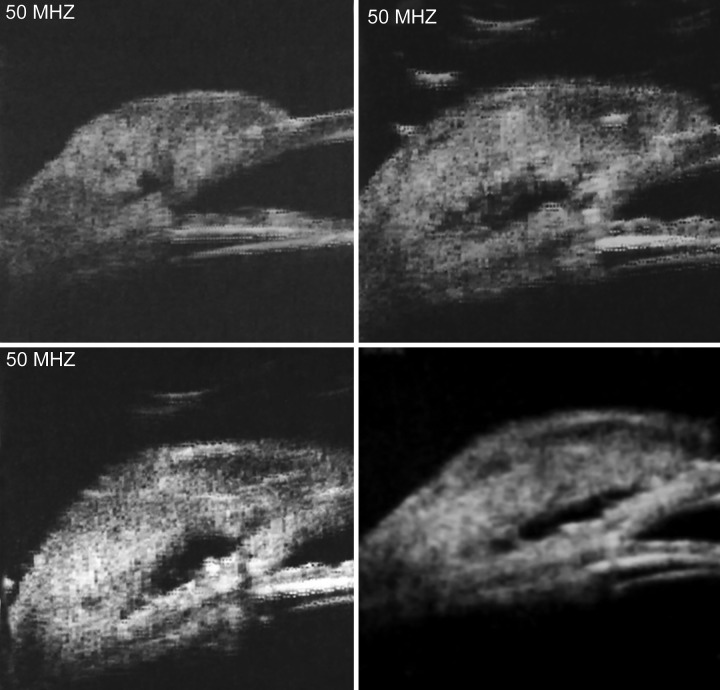
Ultrasound biomicroscopic radial scan revealing a sonoluscent area between deep scleral lamellae “intrascleral lake” (decompression chamber) showing the dolphin head appearance

Ultrasound biomicroscopy was done for both successful and failed cases. The dolphin head has been demonstrated in all the successful cases (Figs 1 and 2). The mouth of the dolphin was formed by the anterior chamber angle, the eye of the dolphin corresponded to the Intrascleral lake, and the vault of the head corresponded to the filtering bleb.

**Figs 3A and B:**
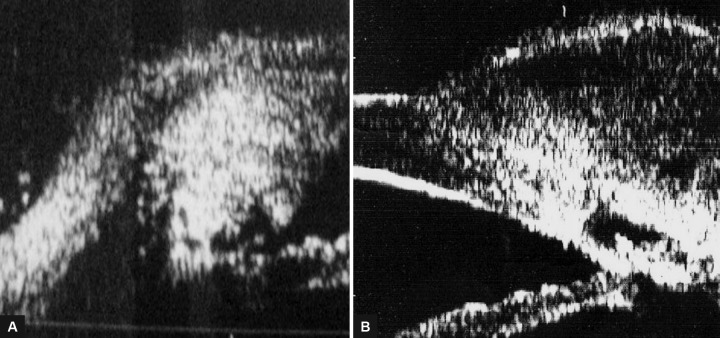
(A) Ultrasound biomicroscopic radial scan revealing the absence of the typical “dolphin head” in failed cases *vs* (B) its presence in successful cases

In failed cases, we noticed that collapse of the intra-scleral lake and or disappearance of the filtering bleb will lead to either a disfigured dolphin head or even its total disappearance (Figs 3A and B).

## DISCUSSION

Ultrasonic biomicroscopy allows structural examination after performing filtering surgery with interpretation similar to that shown in histological sections.^14^ It also provides details of the anterior chamber, ciliary body, and corneoscleral limbus - areas that cannot be examined using other methods in the living eyes.^15^

Ultrasound biomicroscopy is useful in the assessment of anterior chamber angle structures and for evaluating the surgical area after performing various interventions to treat glaucoma. A valuable use of UBM is to assess the filtration area after glaucoma surgeries, penetrating or nonpenetrating, and trying to correlate IOP control to bleb criteria.

Yamamoto et al^16^ used UBM images to further elucidate the internal structure of filtering bleb and were able to establish a new system for classifying filtering blebs based on bleb reflectivity. They also correlated this with IOP control. Blebs were classified into four groups - type L (low reflective) blebs showed good IOP control, with moderate-high bleb height and identifiable microcysts; type H (high reflective), type F (flattened), and type E (encapsulated) were associated with poor IOP control. Other UBM findings reported in literature include progressive reduction in the Intrascleral lake and its eventual collapse.^17^

In our study, we describe a new UBM appearance that could be a clue to successful DS. That appearance of the surgical area can be simulated to a dolphin’s head. The observation of this sign should be taken into consideration during patients’ evaluation and can be considered as a criterion of success as this sign was not elicited in failed cases.

Our study is a short-term one that describes a characteristic appearance of the surgical area during the early postoperative period (3-6 months). Longer follow-ups with serial imaging are required to describe a change in this morphology with time. The present study is limited by the relatively small sample size which can be attributed to the fewer cases which undergo DS and also a relatively short follow-up period.

## CONCLUSION

Presence of a dolphin head configuration in UBM images is suggested to be an indicator of successful surgery in patients undergoing DS.

Financial Disclosure: The authors have no financial interests in the material discussed in this work.
